# Epidemiology and Outcomes of Invasive Candidiasis Due to Non*-albicans* Species of *Candida* in 2,496 Patients: Data from the Prospective Antifungal Therapy (PATH) Registry 2004–2008

**DOI:** 10.1371/journal.pone.0101510

**Published:** 2014-07-03

**Authors:** Michael A. Pfaller, David R. Andes, Daniel J. Diekema, David L. Horn, Annette C. Reboli, Coleman Rotstein, Billy Franks, Nkechi E. Azie

**Affiliations:** 1 JMI Laboratories, North Liberty, Iowa, United States of America; 2 Department of Pathology, University of Iowa, Iowa City, Iowa, United States of America; 3 Department of Medicine, University of Wisconsin, Madison, Wisconsin, United States of America; 4 David Horn LLC, Doylestown, Pennsylvania, United States of America; 5 Department of Medicine, Cooper Medical School of Rowan University, Camden, New Jersey, United States of America; 6 Division of Infectious Diseases, Department of Medicine, University of Toronto, Toronto, Ontario, Canada; 7 Astellas Scientific and Medical Affairs, Northbrook, Illinois, United States of America; New Jersey Medical School, Rutgers University, United States of America

## Abstract

This analysis describes the epidemiology and outcomes of invasive candidiasis caused by non-*albicans* species of *Candida* in patients enrolled in the Prospective Antifungal Therapy Alliance (PATH Alliance) registry from 2004 to 2008. A total of 2,496 patients with non-*albicans* species of *Candida* isolates were identified. The identified species were *C. glabrata* (46.4%), *C. parapsilosis* (24.7%), *C. tropicalis* (13.9%), *C. krusei* (5.5%), *C. lusitaniae* (1.6%), *C. dubliniensis* (1.5%) and *C. guilliermondii* (0.4%); 111 infections involved two or more species of *Candida* (4.4%). Non-*albicans* species accounted for more than 50% of all cases of invasive candidiasis in 15 of the 24 sites (62.5%) that contributed more than one case to the survey. Among solid organ transplant recipients, patients with non-transplant surgery, and patients with solid tumors, the most prevalent non-*albicans* species was *C. glabrata* at 63.7%, 48.0%, and 53.8%, respectively. In 1,883 patients receiving antifungal therapy on day 3, fluconazole (30.5%) and echinocandins (47.5%) were the most frequently administered monotherapies. Among the 15 reported species, 90-day survival was highest for patients infected with either *C. parapsilosis* (70.7%) or *C. lusitaniae* (74.5%) and lowest for patients infected with an unknown species (46.7%) or two or more species (53.2%). In conclusion, this study expands the current knowledge of the epidemiology and outcomes of invasive candidiasis caused by non-*albicans* species of *Candida* in North America. The variability in species distribution in these centers underscores the importance of local epidemiology in guiding the selection of antifungal therapy.

## Introduction

Candidemia and other forms of invasive candidiasis (IC; defined as candidemia or infection involving normally sterile sites) are unquestionably the most prevalent of the invasive mycoses worldwide [1], [2]. More than 30 species of *Candida* have been reported to cause IC [3]–[6]. *Candida albicans* is the most common species encountered in most settings [7]. Other species include *C. glabrata*, *C. parapsilosis*, *C. tropicalis*, *C. krusei*, *C. lusitaniae*, *C. guilliermondii,* and several other infrequently isolated species [4], [8]. In addition, the use of molecular identification methods has resulted in the discovery of new species within the larger species complexes (e.g. *C. dubliniensis* within the *C. albicans* complex, *C. fermentati* within the *C. guilliermondii* complex, and *C. nivariensis* and *C. bracarensis* within the *C. glabrata* clade) [9]–[11].

Longitudinal surveillance studies from individual institutions, cities, countries, and broad geographic regions have documented the emergence of the various non-*albicans Candida* (N-CA) species as well as their potential to develop antifungal resistance [3], [6], [12]–[21]. Resistance to fluconazole and echinocandins has been shown to be more common in N-CA species compared with *C. albicans* isolates in a population based laboratory study [13], and is in part due to N-CA species that are inherently resistant to antifungals, such as *C. krusei* to fluconazole [13] and the greater propensity of species such as *C. glabrata* to develop antifungal resistance [22]. Population-based surveillance of candidemia in the United States (US) conducted by the Centers for Disease Control and Prevention found that the incidence of candidemia due to *C. parapsilosis* and *C. glabrata* increased two- and four-fold, *respectively,* between 1992–1993 and 2008–2011 [13]. The emergence of *C. glabrata* has been reported by other investigators [23]–[27]; however, a recent systematic review by Falagas et al. [7] reported that the species distribution may differ considerably across different geographic regions. Local epidemiological data continue to be of major importance in guiding empirical antifungal therapy in patients with a high probability of developing candidemia [7], [28]–[30].

The Prospective Antifungal Therapy (PATH) Alliance registry was established in 2004, with 25 tertiary care medical centers in the US and Canada prospectively entering consecutive invasive fungal infections (IFIs) into a central database, in an effort to provide a more detailed description of the epidemiology and outcomes of IFIs in North America [31], [32]. Previously, we provided an overview of 3,648 cases of candidemia from the PATH Alliance database for the time period from July 1, 2004 to December 31, 2008 [8]. In the present report, we provide further analysis of the epidemiology, treatment, and outcomes of 2,496 cases of IC due to N-CA species in pediatric and adult patients in the PATH Alliance registry. In addition to broad geographic trends in species distribution, we also examined the species distribution in each of the individual institutions that have provided isolates for each of the 5 years of the study. The latter analysis is to emphasize the importance of local versus regional epidemiologic data, diversity of patient groups affected, and the potential of such information to impact on empiric antifungal therapy.

## Methods

The PATH Alliance registry is a sentinel surveillance network comprising 23 medical centers in the US and two in Canada, which collected data on patients with IFIs. Among the US centers, seven were located in the Northeast region (1,985 patients), seven were located in the South (1,483 patients), six were located in the Midwest (906 patients), and three were located in the West (387 patients). The data collection methodology has previously been described in detail [32]. Briefly, patient information was collected prospectively using a detailed electronic case report form for 12 weeks after diagnosis until the patients died or were lost to follow-up. Information collected included patient baseline demographic characteristics, underlying disease, type of transplant, use of corticosteroids and other immunosuppressive therapies, absolute neutrophil count, infecting *Candida* spp., infection site, and antifungal and adjunctive therapies. Approval for data collection was provided by the institutional review boards of all centers (listed in the Acknowledgements) involved in the study. Patients provided written and informed consent prior to data collection. All data were captured, transferred, and analyzed based on anonymized patient identifiers. *Candida* cultures and histologic specimens were processed, isolated, and identified to the species level at each institution using methods routinely employed at that site. Candidemia was defined as the isolation of *Candida* spp. from blood cultures and other forms of IC (proven or probable) were defined according to the European Organization for Research and Treatment of Cancer/Invasive Fungal Infections Cooperative Group and the National Institute of Allergy and Infectious Diseases Mycoses Study Group criteria [33]. In the present analysis all (100%) cases were proven infections.

The day an IFI was clinically diagnosed using culture or pathology, and first reported to the treating physician was designated as day 1. Descriptive analyses were used for baseline characteristics and subgroup analyses (*Candida* spp. and treatment cohorts). Descriptive survival analyses were performed based on the whole patient group. The survival distribution function was estimated using the Kaplan–Meier method. Patients who were lost to follow-up prior to the week 12 assessment were censored on the day of their last activity, as documented in the database.

## Results and Discussion

Among the 6,845 patients with completed case reports of IFIs, 5,036 (73.6%) patients with candidemia or other forms of IC were identified by the PATH Alliance registry, 2,496 (49.6%) of which were cases of IC due to N-CA species. Of these patients, 2,385 (95.6%) were infected with a single species and 111 (4.4%) were infected with two or more N-CA species. The majority of cases were isolated from blood (n = 2147; 86.0%), while 400 (16.0%) were detected from the abdomen, 35 (1.4%) from the lung, and 34 (1.4%) each from skeleton and skin/soft tissue. N-CA species were also isolated from the CNS (n = 19; 0.8%), heart (n = 14; 0.6%), tracheobronchial mucosa (n = 13; 0.5%), eye (n = 4; 0.2%), sinus (n = 3; 0.1%), and other locations (n = 29; 1.2%). In addition, 226 (9.1%) cases were taken from multiple organs and blood, while 14 (0.6%) were isolated from multiple organs but not blood.

### Isolation of N-CA species by geographic region

In total, 2,496 patients with N-CA isolates were identified (Table 1). Among the 15 different N-CA species identified, seven accounted for 94.1% of infections (Table 1). The proportion of all cases of IC caused by N-CA species ranged from 40.1% in the West to 57.0% in the South. Non-*albicans* species accounted for >50% of all cases of IC in 15 of the 24 sites (62.5%) that contributed more than one case to the survey (Tables 1 and S1). Variation in the frequency of N-CA species was considerable among different centers within most regions i.e., Northeast (range 31.0–67.3%), South (range 29.1–66.4%), Midwest (range 44.4–56.6%), and West (range 32.1–50.6%), with the exception of Canada (range 47.9–48.4%).

**Table 1:** Variation in frequency of non-*albicans* species of *Candida* by geographic region.

	Total no. (%) of patients with N-CA species											
Region/hospital*	All patients n = 5036(100%)	All N-CA n = 2496 (49.6%)	*C. glabrata* n = 1159 (23.0%)	*C. parapsilosis* n = 616(12.2%)	*C. tropicalis* n = 347(6.9%)	*C. kruseI* n = 138(2.7%)	*C. lusitaniae* n = 41 (0.8%)	*C. dubliniensis* n = 38 (0.8%)	*C. guilliermondii* n = 9 (0.2%)	Other†n = 22(0.4%)	Unknownn = 15(0.3%)	Multiple species§ n = 111 (2.2%)
Canada	275	132 (48.0)	53 (19.3)	34 (12.4)	24 (8.7)	12 (4.4)	1 (0.4)	1 (0.4)				7 (2.5)
Northeast	1985	923 (46.5)	427 (21.5)	259 (13.0)	129 (6.5)	31 (1.6)	11 (0.6)	15 (0.8)	4 (0.2)	7 (0.4)		37 (1.9)
South	1483	845 (57.0)	365 (24.6)	203 (13.7)	144 (9.7)	59 (4.0)	1.5 (1.0)	4 (0.3)	1 (0.1)	4 (0.3)		44 (3.0)
Midwest	906	441 (48.7)	244 (26.9)	90 (9.9)	37 (4.1)	25 (2.8)	8 (0.9)	6 (0.7)	1 (0.1)	9 (1.0)	4 (0.4)	17 (1.9)
West	387	155 (40.1)	70 (18.1)	30 (7.8)	13 (3.4)	11 (2.8)	6 (1.6)	12 (3.1)	3 (0.8)	2 (0.5)	2 (0.5)	6 (1.6)

*hospitals designated by the letter ‘H’; and a number: H1, H2 etc.

† other species includes: *C. kefyr* (nine isolates), *C. famata* (four isolates), *C. rugosa* (three isolates), *C. utilis* (two isolates) and one isolate each of *C. fennica*, *C. fermentati*, *C. lipolytica*, and *Torulopsis* spp.

§ multiple species include *C. parapsilosis+C. glabrata* (n = 30), *C. tropicalis+C. glabrata* (n = 21), *C. krusei+C. glabrata* (n = 8), *C. dubliniensis+C. glabrata* (n = 3), *C. lusitaniae+C. glabrata* (n = 4), other *Candida* spp.*+C. glabrata* (n = 3), unknown *Candida spp.+C. glabrata* (n = 3), *C. guilliermondii+C. glabrata* (n = 2), *C. parapsilosis+C. krusei* (n = 5), *C. lusitaniae+C. krusei* (n = 2), *C. tropicalis+C. dubliniensis* (n = 1), *C. tropicalis+C. guilliermondii* (n = 1), *C. tropicalis+C. krusei* (n = 4), other *Candida* spp.+*C.* guilliermondii (n = 1), unknown *Candida* spp.+C. *dubliniensis* (n = 1), *C. glabrata+C. krusei+C. lusitaniae* (n = 1), *C. dubliniensis+C. glabrata+C. guilliermondii* (n = 1).

The rank order of the seven most frequently encountered N-CA species was *C. glabrata>C. parapsilosis>C. tropicalis>C. krusei>C. lusitaniae>C. dubliniensis>C. guilliermondii* in 13 of the 24 sites (54.2%) that contributed more than one case to the survey. *C. glabrata* was the most common of the N-CA species, accounting for 49.4% of N-CA infections (1,159 single species infections and 84 mixed infections [*C. glabrata* plus one other N-CA species]) and was the most frequently isolated species in 20 of the 24 centers (82.5%) that contributed more than one case to the survey. *C. parapsilosis* was the most common N-CA species in four centers, two in the Northeast, one in the Midwest and one in the South. *C. tropicalis* predominated in a cancer center in the South (Tables 1 and S1), whereas *C. krusei*, usually fourth in rank order among N-CA species in most surveys [2], ranked second or third in seven centers. The ‘cryptic’ species *C. dubliniensis* accounted for only 1.5% of all N-CA species (38 of 2,496) in this survey; however, it was detected in 10.3% of N-CA infections in center H23 (West). Differentiation of *C. dubliniensis* from *C. albicans* may reflect the intensity with which the various centers pursue accurate species identification of N-CA [20].

### Baseline patient characteristics

The mean age of patients infected with N-CA species was 53.3 years (standard deviation ±20.0 years) and 52.4% were male (Table 2). Most of the patients were Caucasian (64.6%), followed by African-American (22.1%). Patients had often received antifungal agents as either prophylaxis or empirical therapy within 30 days prior to diagnosis of IC; fluconazole (n = 798, 32.0%) and the echinocandins (n = 363, 14.5%) were the most commonly prescribed antifungal agents (Table 3). The patient groups with the highest proportion of prior exposure to echinocandins were those infected with *C. guilliermondii* (22.2%), followed by those infected with *C. krusei* (21.0%), multiple *Candida* species (19.8%), and *C. tropicalis* (15.0%). Prior therapy with fluconazole was highest in those patients infected with *C. krusei* (60.9%), *C. glabrata* (37.4%), and those with multiple *Candida* species (27.0%). Multiple co-morbidities were common: 52.0% (n = 1297) of patients had a concurrent bacterial infection, 33.6% (n = 838) had diabetes, 32.9% (n = 820) had a malignancy (either hematologic or solid tumor), and 33.9% (n = 845) had undergone a non-transplant-related surgical procedure during hospitalization (Table 4).

**Table 2:** Baseline characteristics.

Parameter	All N-CA	*C. glabrata*	*C. parapsilosis*	*C. tropicalis*	*C. krusei*	*C. lusitaniae*	*C. dubliniensis*	*C. guilliermondii*	Other*	Unknown	Multiple species†
N	2496	1159	616	347	138	*41*	38	9	22	15	111
Age (years), mean (%)	53.3 (20.0)	57.3 (16.7)	47.1 (23.2)	54.3 (20.1)	50.2 (18.7)	46.3 (28.4)	46.1 (15.0)	49.9 (20.2)	47.9 (21.6)	58.9 (12.1)	52.0 (21.4)
Age group (years), n (%)											
<1	45 (1.8)	2 (0.2)	35 (5.7)	1 (0.3)		6 (14.6)					1 (0.9)
1–9	70 (2.8)	7 (0.6)	35 (5.7)	16 (4.6)	2 (1.4)	2 (4.9)			2 (9.1)		6 (5.4)
10–19	56 (2.2)	18 (1.6)	19 (3.1)	5 (1.4)	9 (6.5)	2 (4.9)		1 (11.1)	1 (4.5)		1 (0.9)
20–29	141 (5.6)	47 (4.1)	47 (7.6)	16 (4.6)	14 (10.1)	1 (2.4)	6 (15.8)		2 (9.1)		8 (7.2)
30–39	217 (8.7)	81 (7.0)	63 (10.2)	35 (10.1)	11 (8.0)	4 (9.8)	8 (21.1)	1 (11.1)	2 (9.1)	1 (6.7)	11 (9.9)
40–49	373 (14.9)	174 (15.0)	93 (15.1)	51 (14.7)	18 (13.0)	3(7.3)	9 (23.7)	3 (33.3)	3 (13.6)	2 (13.3)	17 (15.3)
50–59	559 (22.4)	285 (24.6)	119 (19.3)	66 (19.0)	39 (28.3)	4 (9.8)	9 (23.7)	2 (22.2)	4 (18.2)	5 (33.3)	26 (23.4)
60–69	517 (20.7)	279 (24.1)	104 (16.9)	73 (21.0)	26 (18.8)	7 (17.1)	2 (5.3)		5 (22.7)	4 (26.7)	17 (15.3)
70–80	373 (14.9)	181 (15.6)	78 (12.7)	58 (16.7)	16 (11.6)	11 (26.8)	4 (10.5)	2 (22.2)	3 (13.6)	2 (13.3)	18 (16.2)
Males, n (%)	1309 (52.4)	544 (46.9)	358 (58.1)	202 (58.2)	74 (53.6)	26 (63.4)	25 (65.8)	7 (77.8)	12 (54.5)	8 (53.3)	53 (47.7)
Ethnicity, n (%)											
White	1613 (64.6)	789 (68.1)	376 (61.0)	192 (55.3)	97 (70.3)	30 (73.2)	28 (73.7)	7 (77.8)	18 (81.8)	13 (86.7)	63 (56.8)
Black	551 (22.1)	241 (20.8)	146 (23.7)	95 (27.4)	19 (13.8)	8 (19.5)	8 (21.1)	1 (11.1)	3 (13.6)	1 (6.7)	29 (26.1)
Hispanic	86 (3.4)	32 (2.8)	24 (3.9)	17 (4.9)	6 (4.3)	1 (2.4)	1 (2.6)		1 (4.5)		4 (3.6)
Asian	42 (1.7)	11 (0.9)	10 (1.6)	11 (3.2)	4 (2.9)	1 (2.4)		1 (11.1)		1 (6.7)	3 (2.7)
Other/unknown	204 (8.2)	86 (7.4)	60 (9.7)	32 (9.2)	12 (8.7)	1 (2.4)	1 (2.6)				12 (10.8)

*other species includes: *C. kefyr* (nine isolates), *C. famata* (four isolates), *C. rugosa* (three isolates), *C. utilis* (two isolates) and one isolate each of *C. fennica*, *C. fermentati*, *C. lipolytica*, and *Torulopsis* spp.

† multiple species include *C. parapsilosis+C. glabrata* (n = 30), *C. tropicalis+C. glabrata* (n = 21), *C. krusei+C. glabrata* (n = 8), *C. dubliniensis+C. glabrata* (n = 3), *C. lusitaniae+C. glabrata* (n = 4), other *Candida* spp.*+C. glabrata* (n = 3), unknown *Candida spp.+C. glabrata* (n = 3), *C. guilliermondii+C. glabrata* (n = 2), *C. parapsilosis+C. krusei* (n = 5), *C. lusitaniae+C. krusei* (n = 2), *C. tropicalis+C. dubliniensis* (n = 1), *C. tropicalis+C. guilliermondii* (n = 1), *C. tropicalis+C. krusei* (n = 4), other *Candida* spp.+*C. guilliermondii*.

**Table 3:** Prior antifungal therapy.

Prior antifungal therapy, n (%)	All N-CA	*C. glabrata*	*C. parapsilosis*	*C. tropicalis*	*C. krusei*	*C. lusitaniae*	*C. dubliniensis*	*C. guilliermondii*	Other*	Unknown	Multiple species†
Echinocandin	363 (14.5)	158 (13.6)	86 (14.0)	52 (15.0)	29 (21.0)	4 (9.8)	5 (13.2)	2 (22.2)	3 (13.6)	2 (13.3)	22 (19.8)
Amphotericin B	122 (4.9)	38 (3.3)	47 (7.6)	19 (5.5)	9 (6.5)	1 (2.4)	1 (2.6)	1 (11.1)	2 (9.1)		4 (3.6)
Fluconazole	798 (32.0)	434 (37.4)	148 (24.0)	76 (21.9)	84 (60.9)	9 (22.0)	5 (13.2)	2 (22.2)	5 (22.7)	5 (33.3)	30 (27.0)
Voriconazole	125 (5.0)	62 (5.3)	16 (2.6)	18 (5.2)	18 (13.0)	2 (4.9)	2 (5.3)	1 (11.1)	1 (4.5)	1 (6.7)	4 (3.6)
Itraconazole	16 (0.6)	3 (0.3)	1 (0.2)	4 (1.2)	3 (2.2)	1 (2.4)	1 (2.6)	1 (11.1)	1 (4.5)	1 (6.7)	
Posaconazole	8 (0.3)	3 (0.3)		1 (0.3)	2 (1.4)	1 (2.4)					1 (0.9)
Flucytosine	3 (0.1)	2 (0.2)			1 (0.7)						
Blinded	5 (0.2)	2 (0.2)	2 (0.3)	1 (0.3)							
Other	160 (6.4)	99 (8.5)	33 (5.4)	10 (2.9)	8 (5.8)	2 (4.9)			3 (13.6)		5 (4.5)

*other species includes: *C. kefyr* (nine isolates), *C. famata* (four isolates), *C. rugosa* (three isolates), *C. utilis* (two isolates) and one isolate each of *C. fennica*, *C. fermentati*, *C. lipolytica*, and *Torulopsis* spp.

† multiple species include *C. parapsilosis+C. glabrata* (n = 30), *C. tropicalis+C. glabrata* (n = 21), *C. krusei+C. glabrata* (n = 8), *C. dubliniensis+C. glabrata* (n = 3), *C. lusitaniae+C. glabrata* (n = 4), other *Candida* spp.*+C. glabrata* (n = 3), unknown *Candida spp.+C. glabrata* (n = 3), *C. guilliermondii+C. glabrata* (n = 2), *C. parapsilosis+C. krusei* (n = 5), *C. lusitaniae+C. krusei* (n = 2), *C. tropicalis+C. dubliniensis* (n = 1), *C. tropicalis+C. guilliermondii* (n = 1), *C. tropicalis+C. krusei* (n = 4), other *Candida* spp.+*C. guilliermondii.*

**Table 4:** Patient category and underlying conditions.

Patient category, n (%)	All N-CA	*C. glabrata*	*C. parapsilosis*	*C. tropicalis*	*C. krusei*	*C. lusitaniae*	*C. dubliniensis*	*C. guilliermondii*	Other*	Unknown	Multiple species†
General medicine	1723 (69.0)	807 (69.6)	432 (70.1)	235 (67.7)	87 (63.0)	25 (61.0)	26 (68.4)	4 (44.4)	14 (63.6)	10 (66.7)	83 (74.8)
Hematologic malignancy	381 (15.3)	126 (10.9)	61 (9.9)	77 (22.2)	77 (55.8)	6 (14.6)	10 (26.3)	4 (44.4)	8 (36.4)	4 (26.7)	8 (7.2)
Stem cell transplantation	139 (5.6)	56 (4.8)	24 (3.9)	13 (3.7)	32 (23.2)	1 (2.4)	3 (7.9)	2 (22.2)	2 (9.1)	2 (13.3)	4 (3.6)
HIV/AIDS	49 (2.0)	20 (1.7)	8 (1.3)	8 (2.3)	4 (2.9)		5 (13.2)				4 (3.6)
NICU	29 (1.2)	1 (0.1)	24 (3.9)			4 (9.8)					
Solid organ transplant	273(10.9)	174 (15.0)	41 (6.7)	22 (6.3)	12 (8.7)	5 (12.2)			4 (18.2)	3 (20.0)	12 (10.8)
Solid tumor	439 (17.6)	236 (20.4)	80 (13.0)	52 (15.0)	19 (13.8)	12 (29.3)	4 (10.5)	3 (33.3)	6 (27.3)	3 (20.0)	24 (21.6)
Surgical (non-transplant)	845 (33.9)	406 (35.0)	225 (36.5)	101 (29.1)	30 (21.7)	13 (31.7)	8 (21.1)	2 (22.2)	5 (22.7)	6 (40.0)	49 (44.1)
**Main underlying conditions, n (%)**											
Diabetes	838 (33.6)	434 (37.4)	188 (30.5)	111 (32.0)	29 (21.0)	9 (22.0)	9 (23.7)	3 (33.3)	6 (27.3)	10 (66.7)	39 (35.1)
Neutropenia	259 (10.4)	74 (6.4)	37 (6.1)	55 (15.9)	61 (44.2)	4 (9.8)	7 (18.4)	4 (44.4)	6 (27.3)	4 (26.7)	7 (6.3)
GVHD	22 (0.9)	13 (1.1)	3 (0.5)	1 (0.3)	2 (1.4)		1 (2.6)		1 (4.5)	1 (6.7)	
Corticosteroids	288 (11.5)	99 (8.5)	41 (6.8)	55 (15.9)	61 (44.2)	5 (12.2)	8 (21.1)	4 (44.4)	6 (27.3)	2 (13.3)	7 (6.3)
Inherited immunodeficiency disorder	12 (0.5)	2 (0.2)	6 (1.0)	2 (0.6)		1 (2.4)	1 (2.6)				
Concomitant infection, n (%)											
CMV	49 (2.0)	21 (1.8)	8 (1.3)	6 (1.7)	10 (7.2)			1 (11.1)		2 (13.3)	1 (0.9)
Bacterial	1297 (52.0)	619 (53.4)	313 (50.8)	176 (50.7)	54 (39.1)	24 (58.5)	22 (57.9)	2 (22.2)	12 (54.5)	8 (53.3)	67 (60.4)

*Other species include: *C. kefyr* (nine isolates), *C. famata* (four isolates), *C. rugosa* (three isolates), *C. utilis* (two isolates) and one isolate each of *C. fennica*, *C. fermentati*, *C. lipolytica*, and *Torulopsis* spp.

† multiple species include *C. parapsilosis*+*C. glabrata* (n = 30), *C. tropicalis*+*C. glabrata* (n = 21), *C. krusei*+*C. glabrata* (n = 8), *C. dubliniensis*+*C. glabrata* (n = 3), *C. lusitaniae*+*C. glabrata* (n = 4), other *Candida* spp.+*C. glabrata* (n = 3), unknown *Candida* spp.+*C. glabrata* (n = 3), *C. guilliermondii*+*C. glabrata* (n = 2), *C. parapsilosis*+*C. krusei* (n = 5), *C. lusitaniae*+*C. krusei* (n = 2), *C. tropicalis*+*C. dubliniensis* (n = 1), *C. tropicalis*+*C. guilliermondii* (n = 1), *C. tropicalis*+*C. krusei* (n = 4), other *Candida* spp.+*C. guilliermondii* (n = 1), unknown *Candida* spp.+*C. dubliniensis* (n = 1), *C. glabrata*+*C. krusei*+*C. lusitaniae* (n = 1), *C. dubliniensis*+*C. glabrata*+*C. guilliermondii* (n = 1).

CMV = cytomegalovirus; GVHD = graft versus host disease; NICU = neonatal intensive care unit.

### *Candida* epidemiology

Among the 2,496 isolates of N-CA species, *C. glabrata* was the predominant species (n = 1159, 46.4%); however, the proportion of *C. glabrata* isolates varied considerably among the participating hospitals (range 5.6–64.3%; Tables 1 and S1). Infections with *C. glabrata* were most prominent in patients with a solid organ transplant (SOT; 63.7% of reported SOT patients with infections), and in those with solid tumors (53.8% of solid tumor patients with infections, Table 4). In contrast, *C. glabrata* was a rare cause of IC in the neonatal intensive care unit (NICU; 3.4% of patients). Patients with *C. glabrata* infections were generally older (mean age, 57.3 years), had diabetes (37.4%), and had experienced prior exposure to azoles (n = 502, 43.3%) or echinocandins (n = 158, 13.6%). Notably, resistance to the echinocandins is emerging in *C. glabrata* with co-resistance to both azoles and echinocandins in some medical centers [12], [13], [20]. Analysis of 313 isolates of *C. glabrata* over a 10-year period in one medical center demonstrated an increase in resistance to echinocandins from 4.9% to 12.3%, while resistance to fluconazole increased from 18% to 30% [12]. Notably, 14.1% of fluconazole-resistant isolates were resistant to at least one echinocandin. Prior therapy with azoles and echinocandins was shown to predict resistance to each class of antifungal agents in patients infected with *C. glabrata* [12].

*C. parapsilosis* was the most frequently isolated N-CA species in four centers (H1 [Midwest], H7 [Northeast], H17 [South], and H25 [Northeast]) and the second most common in 18 centers, accounting for 24.7% (n = 616) of all patients with N-CA isolates (range 6.3–38.9%; Tables 1 and S1). *C. parapsilosis* was responsible for infections in younger individuals (61.8% of patients in aged 1–19 years) (Table 2) and accounted for 82.8% of N-CA IC in patients in the NICU (data not shown). *C. parapsilosis* has been observed to be a significant neonatal pathogen. In a meta-analysis of neonatal literature, 33.5% of all neonatal *Candida* infections were caused by *C. parapsilosis* [34]. Furthermore, an analysis of bloodstream infection data in neonates collected between 1995 and 2004, from 128 NICUs in the United States, showed *C. parapsilosis* was the cause of 33.7% of all *Candida* infections and for 80.2% of all N-CA infections [35]. Patients with *C. parapsilosis*-associated IC were likely to have had recent non-transplant surgery (36.5%) and were unlikely to have experienced prior antifungal therapy or to have received corticosteroid therapy (Tables 3 and 4). The decreased susceptibility of *C. parapsilosis* to the echinocandins is a well-known concern [29], with some centers reporting a decreased prevalence of *C. albicans* in favor of *C. parapsilosis* as a cause of IC in patients pre-exposed to echinocandins [36], [37].

There is an ongoing debate on whether echinocandins are appropriate for the treatment of infections due to the *C. parapsilosis* complex [38]. Indeed Andes et al [39] found in a patient-level review of clinical trials data that the echinocandins were the least effective therapy for infections due to the *C. parapsilosis* complex. Conversely, a recent study by Fernandez-Ruiz et al [40] found that the initial use of an echinocandin for treatment of IC due to *C. parapsilosis* did not result in a worse outcome compared to that seen with the azoles. Irrespective of the choice of therapy for infection with *C. parapsilosis,* the early removal of central venous catheters remains an important principle in the management of candidemia caused by this species [29].

*C. tropicalis* accounted for 13.9% of all N-CA infections (range, 3.0–37.0%; Table 1), and was the third most frequently isolated N-CA species in 17 centers (68.0%). Patients with IC due to *C. tropicalis* often had a hematologic malignancy (22.2%), were neutropenic (15.9%), and had received corticosteroids (15.9%; Table 4). *C. tropicalis* was an uncommon cause of infection in the pediatric age group (12.8% of patients with infections aged <1–19 years) and was not isolated from patients in the NICU (Table 2). Prior exposure to antifungal agents was uncommon in patients infected with *C. tropicalis,* which is generally quite susceptible to both azoles and echinocandins [13], [21], and the use of these agents has been associated with a decrease in the incidence of blood stream infections (BSI) due to *C. tropicalis* in US cancer centers [41], [42]. Reports from Taiwan have highlighted the emergence of fluconazole resistance in *C. tropicalis* from a variety of specimen types [43], and these data are supported by global surveillance data showing that the highest rates of resistance among *C. tropicalis* to fluconazole were seen among isolates from the Asia-Pacific region [44].

Overall, *C. krusei* caused 5.5% of all N-CA infections (range, 1.1–22.2%; Table 1), and was the fourth most common species detected in 16 centers (64.0%; Table S1.) *C. krusei* was found to cause infections in 23 of the 25 participating centers. Patients infected with *C. krusei* were frequently neutropenic (44.2%), were recipients of a stem cell transplant (23.2%), and suffered from a hematologic malignancy (55.8%). Of the patients with *C. krusei* infection 77.5% had received prior antifungal therapy with azoles, 21.0% had received prior therapy with echinocandins, and 44.2% had been treated with corticosteroids (Tables 3 and 4). *C. krusei* is best known for intrinsic resistance to fluconazole as well as its propensity to emerge in settings where fluconazole is used in prophylaxis [41], [45]. Fortunately, *C. krusei* remains susceptible to both voriconazole and the echinocandins [21].

Although the 11 remaining species collectively accounted for only 4.4% of all N-CA isolates in this registry, there are several that merit attention either because they have been shown to cause clusters of infection in the hospital setting, because they are frequently misidentified by conventional methods or represent ‘cryptic’ species, or because they exhibit decreased susceptibility to one or more antifungal agents and therefore pose a threat in certain settings [4], [14], [46].

*C. lusitaniae* accounted for 1.6% of all N-CA isolates and was reported as a cause of IC in 17 of the 25 participating centers (Table S1). *C. lusitaniae* was responsible for infections in younger patients (mean age, 46.3 years) and 14.6% of infections occurred in patients less than 1 year of age (Table 2). Approximately one-third of patients infected with *C. lusitaniae* suffered from a solid tumor (29.3%) and/or had a surgical procedure during hospitalization (31.7%). *C. lusitaniae* was associated with only one case of IC in stem cell transplant recipients (0.7% of all stem cell transplant recipients with infections) and in patients with a hematologic malignancy (1.6%). Patients infected with *C. lusitaniae* infrequently received prior antifungal therapy or corticosteroids (Table 4). *C. lusitaniae* is often mentioned in the literature as being capable of developing resistance to amphotericin B during the course of therapy and may manifest as breakthrough infection in immunocompromised patients on amphotericin B therapy [4], [47], [48], Atkinson et al. [47] reported that, in contrast to patients with fungemia due to *C. albicans*, patients with BSI caused by *C. lusitaniae* had an increased treatment failure rate when they were treated with an amphotericin B-based regimen (10% versus 38%, respectively; *p* = 0.04). *C. lusitaniae* remains susceptible to both the triazoles and the echinocandins, although acquired resistance to the latter group of agents has been reported [14], [21], [49].

*C. dubliniensis* is a ‘cryptic’ species previously indistinguishable from *C. albicans* [11], [50]. In contrast to the other ‘cryptic’ species of *Candida*, *C. dubliniensis* can now be differentiated from *C. albicans* by non-molecular methods [51]. Although originally strictly associated with oropharyngeal candidiasis, *C. dubliniensis* is now recognized as an uncommon cause of IC [50]. In the PATH Alliance registry, *C. dubliniensis* accounted for 1.5% (n = 38) of all patients with N-CA infections, ranking sixth out of the 15 N-CA species reported (Table 2); 26.3% of all *C. dubliniensis* infections occurred among patients with hematologic malignancies. Of all infections in patients with HIV/AIDS, 10.2% were caused by *C. dubliniensis* (Table 4). In addition, 21.1% of patients infected with *C. dubliniensis* had a surgical procedure during hospitalization. *C. dubliniensis* was not reported to cause infection in pediatric patients, those in the NICU, or in recipients of SOT (Tables 2 and 4). Prior exposure to antifungal agents was uncommon in patients infected with *C. dubliniensis* (Table 3). Although fluconazole resistance was previously reported to be common among isolates of *C. dubliniensis* from cases of oropharyngeal candidiasis in the pre-HAART era [52], subsequent studies have found most isolates to be susceptible to fluconazole, the newer azoles, as well as the echinocandins [14], [53].

Infection with *C. guilliermondii* has been found to be more common among patients with cancer and among patients with prior cardiovascular or intra-abdominal surgery [16], [54]. In the PATH Alliance registry, *C. guilliermondii* accounted for only 0.4% of all N-CA infections and was detected in six of the 25 participating centers (Table S1). *C. guilliermondii* was isolated from only one patient in the pediatric population (0.6% of all infections in the <1–19 years age group) and was not detected in patients in the NICU (Table 2). *C. guilliermondii* caused infections in patients with hematologic malignancies, solid tumors, and those with neutropenia (Table 4). Exposure to corticosteroids was seen in 44.4% of patients with IC due to *C. guilliermondii*. Concomitant bacterial infection was uncommon in patients with *C. guilliermondii* infection as was prior exposure to antifungal agents (Table 3). *C. guilliermondii* is known to exhibit decreased susceptibility to amphotericin B, fluconazole, and the echinocandins [14], [16], [54].

Among the eight ‘other’ species included in the PATH Alliance registry, four merit some discussion: *C. kefyr, C. famata, C. fermentati,* and *C. rugosa* (Tables 1 and 2). Although extremely uncommon, one or more of these ‘other’ species were detected in 12 of the 25 participating centers (Table S1). While some of these species have been designated as ‘emerging’ species, the apparent increased detection of these species may simply reflect the more vigorous efforts to identify clinical isolates of *Candida* to the species level in recent years [6], [20], [46].

*C. kefyr* has been called an ‘emerging’ pathogen in patients with leukemia and neutropenia based on two reports documenting nosocomial BSI and colonization [55], [56]. The reasons for the emergence of *C. kefyr* in the fungal flora of neutropenic patients are not known but may be secondary to exposure to antifungal agents, as some strains of *C. kefyr* exhibit high minimum inhibitory concentrations when tested with amphotericin B [14], [57]. More recently, resistance to echinocandins was documented in a strain emerging during caspofungin treatment [58].

*C. famata* is a rare cause of IC that has recently been shown to exhibit reduced susceptibility to echinocandins and azoles, potentially in the setting of prior antifungal exposure [59]. It should be noted that phenotypic differentiation between *C. famata* and *C. guilliermondii* is very difficult as these two species share many of the same biochemical and morphological features [60], [61]. In a recent analysis of 53 isolates reported to be *C. famata*, Castanheira et al. [61] found that none were *C. famata* when subjected to nucleic acid sequencing and matrix-assisted laser desorption ionization-time of flight mass spectrometry. *C. guilliermondii* and *C. parapsilosis* were the species most frequently misidentified as *C. famata*. These findings support the use of molecular and proteomic methods for species identification, especially when dealing with these very rare N-CA species [20]. Similar concerns surround the identification of the ‘cryptic’ species *C. fermentati*. *C. fermentati* isolates make up a small percentage (8.7%) of the *C. guilliermondii* complex and are only identified using molecular methods at the present time [10]. Notably, both *C.famata* and *C. fermentati* share the same antifungal susceptibility profile with *C. guilliermondii* marked by decreased susceptibility to both echinocandins and azoles [10], [59]. Although conventional phenotypic methods for identification work well for the common species of *Candida*, they are unable to differentiate among cryptic species within any of the species complexes [9], [10] and are not reliable for the identification of less common species where nucleic acid sequencing and proteomic methods are required for accurate identification [20].

*C. rugosa* is notable for causing nosocomial clusters of fungemia [62], [63] and for decreased susceptibility to both azoles and echinocandins [15]. *C. rugosa* was found to be more common as a cause of IC in Latin America (2.7% of all *Candida* spp.) versus other regions of the world (0.1–0.4%) [15].

Candidemia or other forms of IC due to more than one species of *Candida* is uncommon, accounting for 1.6–6% of all cases [64], [65]. IC due to two or more species of N-CA was documented in 2.2% of all cases of IC and 4.4% of all cases of N-CA IC in the present study (Table 1). Polyfungal IC caused by N-CA species was documented in 21 of 25 contributing sites in the PATH Alliance registry. There were two episodes with more than two species identified: one patient infected with *C. glabrata+C. krusei+C. lusitaniae* and one patient infected with *C. dubliniensis+C. glabrata+C. guilliermondii*. *C. glabrata* plus *C. parapsilosis* was the most frequent combination of species accounting for 27.0% of all N-CA polyfungal infections, followed by *C. glabrata* plus *C. tropicalis* (18.9%). Overall *C. glabrata* was involved in 67.6% of the mixed N-CA infections. Polyfungal IC is an uncommon nosocomial entity occurring in non-oncologic patients with multiple co-morbidities and heavy *Candida* colonization [64]. In the PATH Alliance registry, patients infected with multiple N-CA species had frequently undergone a surgical procedure (44.1%) during the hospitalization period (Table 4). Mortality is reported to be similar to that seen with monomicrobial IC, but less than that seen with polymicrobial bacteremia [64]–[66].

### Diagnostic tests

The vast majority of the infections due to N-CA species in the PATH Alliance registry were diagnosed by blood culture (n = 2,133/2,496; 85.5%). An additional 62 infections (2.5%) were diagnosed by histopathological or cytopathological examination of sterile body sites, 283 (11.3%) by culture of deep tissue sites or normally sterile body fluids, and 17 (0.7%) by computed tomographic scan of the abdomen. Notably, tests for detection of fungal antigens or nucleic acids were only employed in 0.6% of cases, suggesting that these newer assays are not well utilized in clinical practice Given the predominance of blood culture as a diagnostic modality, the frequency of IC in this survey is almost certainly an underestimate. It is estimated that approximately 30% of cases of IC are not associated with candidemia [67] and a recent report by Clancy and Nguyen [68] found the sensitivity of ante-mortem blood culture for the diagnosis of autopsy-proven IC was only 38%. The use of non-culture tests as biomarkers may prove to be useful adjuncts to blood culture, and identify some patients who are currently undiagnosed [68]. Current European Society of Clinical Microbiology and Infectious Disease guidelines recommend the β-D-glucan test, the mannan/anti-mannan test (candidemia only), and direct microscopy and histopathology as non-culture tests for diagnosis of IC [69]. PCR tests are also emerging as potential diagnosis tools, but are not recommended currently due to a lack of standardization [69].

### Antifungal therapy

Of the 2,496 patients with IC due to N-CA species, 2,184 (87.5%) received antifungal therapy. A total of 312 patients ostensibly had no treatment, of whom 174 (55.8%) died before treatment was administered, 63 (20.2%) were lost to follow-up, and 75 (24%) lived through to the end of follow-up. Among the 1,883 patients receiving antifungal therapy on day 3 (selected as a representative treatment day), 47.5% received an echinocandin (n = 895), 30.5% received fluconazole (n = 575), and only 7.9% received either a lipid formulation of amphotericin B (n = 136) or amphotericin B deoxycholate (n = 13; Table 5). Lower numbers of patients reported treatment on day 3 as compared to day 1 due to mortality by day 3. Combination or sequential therapy, generally with an echinocandin plus fluconazole, was reported in 45.5% of patients with IC. The majority of patients (n = 1,912; 76.6%) received empiric antifungal therapy on day 1 and 75.4% (n = 1883) received antifungal therapy on day 3 after the positive diagnostic test. The average duration of antifungal therapy post-diagnosis was 21.6 days.

**Table 5:** Antifungal treatment administered by species on infection day 3.

	Total no (%) of patients treated by species										
Antifungal treatment	All N-CA	*C. glabrata*	*C. parapsilosis*	*C. tropicalis*	*C. krusei*	*C. lusitaniae*	*C. dubliniensis*	*C. guilliermondii*	Other*	Unknown	Multiple species†
N	2496	1159	616	347	138	41	38	9	22	15	111
N treated on day 3	1883	846	471	260	114	29	24	8	20	10	101
Monotherapy											
Echinocandin	895 (47.5)	488 (57.7)	145 (30.8)	109 (41.9)	59 (51.8)	7 (24.1)	11 (45.8)	5 (62.5)	11 (55.0)	5 (50.0)	55 (54.5)
Amphotericin B	149 (7.9)	41 (4.8)	58 (12.3)	15 (5.8)	15 (13.2)	3 (10.3)	3 (12.5)	1 (12.5)	4 (20.0)	2 (20.0)	7 (6.9)
Fluconazole	575 (30.5)	224 (26.5)	195 (41.4)	94 (36.2)	11 (9.6)	18 (62.1)	7 (29.2)		3 (15.0)	3 (30.0)	20 (19.8)
Voriconazole	34 (1.8)	13 (105)	9 (1.9)	5 (1.9)	5 (4.4)						2 (2.0)
Posaconazole	2 (0.1)	1 (0.1)			1 (0.9)						
Itraconazole	1 (<0.1)				1 (0.9)						
Blinded§	21 (1.1)	10 (1.2)	4 (0.8)	2 (0.8)	4 (3.5)						1 (1.0)
Combination therapy											
Voriconazole+echinocandin	28 (1.5)	14 (1.7)	3 (0.6)	3 (1.2)	5 (4.4)	1 (3.4)			1 (5.0)		1 (1.0)
Voriconazole+amphotericin B	8 (0.4)	1 (0.1)	5 (1.1)				1 (4.2)	1 (12.5)			
Amphotericin B+echinocandin	27 (1.4)	7 (0.8)	3 (0.6)	7 (2.7)	6 (5.3)		1 (4.2)	1 (12.5)	1 (5.0)		1 (1.0)
Voriconazole+amphotericin B+echinocandin	8 (0.4)	1 (0.1)	2 (0.4)	3 (1.2)	2 (1.8)						
Fluconazole+echinocandin	94 (5.0)	37 (4.4)	29 (6.2)	17 (6.5)	3 (2.6)						8 (7.9)
Other‡	41 (2.2)	9 (1.1)	18 (3.8)	5 (1.9)	2 (1.8)		1 (4.2)				6 (5.9)

*Other species includes: *C. kefyr* (nine isolates), *C. famata* (four isolates), *C. rugosa* (three isolates), *C. utilis* (two isolates) and one isolate each of *C. fennica*, *C. fermentati*, *C. lipolytica*, and *Torulopsis* spp.

† multiple species include *C. parapsilosis+C. glabrata* (n = 30), *C. tropicalis+C. glabrata* (n = 21), *C. krusei+C. glabrata* (n = 8), *C. dubliniensis+C. glabrata* (n = 3), *C. lusitaniae+C. glabrata* (n = 4), other *Candida* spp.*+C. glabrata* (n = 3), unknown *Candida spp.+C. glabrata* (n = 3), *C. guilliermondii+C. glabrata* (n = 2), *C. parapsilosis+C. krusei* (n = 5), *C. lusitaniae+C. krusei* (n = 2), *C. tropicalis+C. dubliniensis* (n = 1), *C. tropicalis+C. guilliermondii* (n = 1), *C. tropicalis+C. krusei* (n = 4), other *Candida* spp.+*C.* guilliermondii (n = 1), unknown *Candida* spp.+C. *dubliensis* (n = 1), *C. glabrata+C. krusei+C. lusitaniae* (n = 1), *C. dubliniensis+C.glabrata+C.guilliermondii* (n = 1).

§ patients in this category were in a blinded clinical trial.

‡ Other combination therapies included lipid amphotericin B +5-fluorocytosine (n = 7), lipid amphotericin B+fluconazole (n = 10), amphotericin B deoxycholate+lipid amphotericin B (n = 1), amphotericin B deoxycholate+fluconazole (n = 2), echinocandins +5-fluorocytosine (n = 1), echinocandins+amphotericin B deoxycholate (n = 9), echinocandins+fluconazole (n = 1), echinocandins+itraconazole (n = 1), fluconazole+blinded (n = 1), fluconazole+voriconazole (n = 8).

The use of each antifungal agent varied considerably with the species of *Candida* isolated from the blood or other normally sterile sites (Table 5). A fungicidal agent such as an echinocandin is recommended for initial treatment in severely compromised or unstable hosts whereas fluconazole is recommended for step-down therapy for patients initially treated with an echinocandin or as primary therapy for patients infected with *C. parapsilosis* [28]–[30]. An echinocandin was used most frequently in patients infected with *C. glabrata* (57.7%), *C. krusei* (51.8%), *C. guilliermondii* (62.5), other N-CA species (55.0%) and multiple N-CA species (54.5%), whereas fluconazole was used in patients infected with *C. parapsilosis* (41.4%) and *C. lusitaniae* (62.1%). Amphotericin B was most often used in the treatment of IC due to *C. parapsilosis* (12.3%), *C. krusei* (13.2%), *C. dubliniensis* (12.5%), and *C. guilliermondii* (12.5%). Combination/sequential therapy with an echinocandin and either voriconazole or amphotericin B was used most often in patients infected with *C. krusei*.

On reviewing the approaches to antifungal therapy reported by the participating centers, several appear to be at odds with current consensus guidelines [28]–[30] and reported susceptibility profiles in the literature [14], [21]. Despite the well-known susceptibility of *C. parapsilosis, C. tropicalis and C. dubliniensis* to fluconazole [21], 30.8%, 41.9% and 45.8% respectively, were treated with an echinocandin on day 3 (Table 5). This apparent deviation may be explained by use in unstable hosts where a fungicidal agent may be desired or by delays in receiving information about species identification and susceptibilities. This tendency was reversed by day 10 (data not shown) suggesting that a transition from an echinocandin to fluconazole was occurring as patients stabilized and/or the species and the antifungal susceptibility profile became known. Likewise, 11 patients infected with *C. krusei*, a well-known fluconazole-resistant species, were treated with fluconazole on day 3 (Table 5). This number decreased to five by day 10 (data not shown); however, fluconazole remains an inappropriate therapy for IC caused by *C. krusei* [29].

The evolution of treatment patterns over time is best seen in the cohort of patients infected with *C. glabrata* (Table 6). This example shows an increased use of an echinocandin and a proportional decrease in the use of fluconazole over time, presumably due to the species identification, availability of antifungal susceptibility results, and response of the patient to antifungal therapy.

**Table 6:** Changes in the treatment of invasive candidiasis due to *C. glabrata* over time.

Antifungal treatment	No. (%) treated			
	Day 1	Day 3	Day 10	Day 30
N	866	846	683	143
Monotherapy				
Echinocandin	409 (47.2)	488 (57.7)	438 (64.1)	86 (60.1)
Amphotericin B	41 (4.7)	41 (4.9)	37 (5.4)	7 (4.9)
Fluconazole	314 (36.3)	224 (26.5)	143 (20.9)	28 (19.6)
Voriconazole	14 (1.6)	13 (1.5)	23 (3.4)	14 (9.8)
Posaconazole	1 (0.1)	1 (0.1)	2 (0.3)	1 (0.7)
Combination therapy				
Voriconazole+echinocandin	9 (1.0)	14 (1.7)	8 (1.2)	2 (1.4)
Voriconazole+amphotericin B	2 (0.2)	1 (0.1)	1 (0.1)	
Amphotericin B+echinocandin	5 (0.6)	7 (0.8)	5 (0.7)	
Voriconazole+amphotericin B+echinocandin		1 (0.1)	1 (0.1)	
Fluconazole+echinocandin	57 (6.6)	37 (4.4)	13 (1.9)	3 (2.1)
Other	6 (0.7)	9 (1.1)	4 (0.6)	1 (0.7)

### Patient outcomes

**Figure 1:**
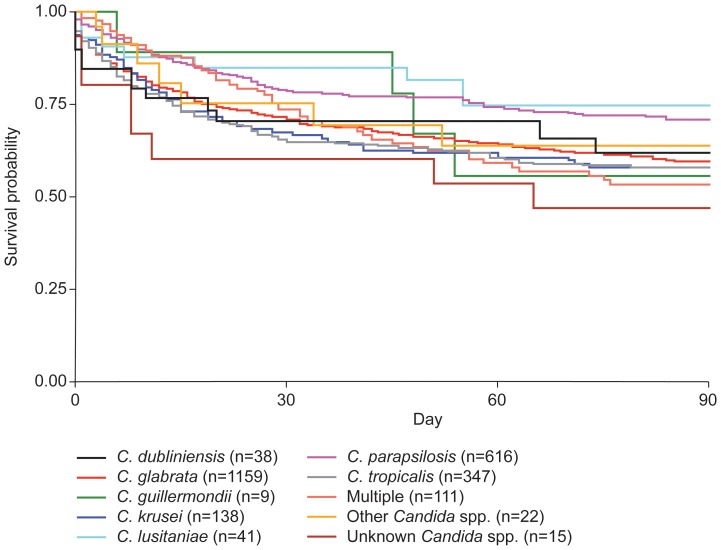
90-day survival stratified by non-*albicans Candida* species.

Patient survival, stratified by *Candida* species, is shown in Figure 1. The 90-day survival rate for all patients was 61.7%, in keeping with other reports [1]. Survival patterns among patients with IC caused by *C. glabrata, C. tropicalis, C. krusei, C. guilliermondii* and *C. dubliniensis* were similar, ranging from 57.7% (*C. krusei*) to 61.4% (*C. dubliniensis*; Figure 1). Survival was lowest for patients infected with multiple N-CA species (53.1%), and highest for those infected with *C. parapsilosis* (70.7%) and *C. lusitaniae* (74.5%). In a similar multicenter observation study of patients with *Candida* infection, a 3-month mortality rate of 35% was observed for all N-CA infections in patients >13 years of age, with the highest mortality rate observed for *C. tropicalis* (48%) and the lowest for *C. parapsilosis* (24%) [65]. This is in contrast with the current analysis in which *C. krusei* had the lowest survival rate and *C. lusitaniae* the highest. *C. parapsilosis* was associated with high survival in both studies.

## Conclusions

First and foremost, these results document the emergence of N-CA species collectively surpassing *C. albicans* in most, but not all, sentinel tertiary care medical centers in North America. Whereas several studies document a clear increase in the incidence of N-CA species such as *C. glabrata, C. parapsilosis* and *C. tropicalis* [7], [13], [23], [25], [36], [41]–[43], [45], the apparent “emergence” of less common species may be due to a greater emphasis on species identification as a guide to initial treatment choices [28]–[30]. The variability in species distribution in these centers, coupled with the fact that several N-CA species may express resistance to one or more antifungal agent, underscores the importance of local epidemiology in guiding the selection of empirical antifungal therapy. The results of this survey both confirm and extend our understanding of IC due to N-CA species in North American tertiary care medical centers. Common themes include the complicated nature of the patient population, a high frequency of concomitant bacterial infection, prior exposure to antifungal agents and immunosuppressive agents (corticosteroids), and high rates of diabetes and prior surgery. Exposures specific to different species were also confirmed: prior antifungal therapy with *C. glabrata* and *C. krusei*; neutropenia with *C. krusei* and *C. guilliermondii*; hematologic malignancies and stem cell transplantation with *C. krusei* and *C. guilliermondii*; older age with *C. glabrata* and younger age (NICU and pediatric patients) with *C. parapsilosis* and prior surgery and solid organ transplantation with *C. glabrata*. The broad range of N-CA species detected in these centers is a testament to an increased emphasis on species identification as an aid to optimizing antifungal therapy and allows us to comment on the propensity of the less common species for specific patient niches. Whereas the rank order of species was the same in 54.2% of participating sites, there was considerable variation in the frequency of some species from center to center. *C. glabrata* was the predominant species in most centers but the frequency ranged from 5.6–64.3% of all N-CA cases of IC across the 25 centers. We confirm the fact that *C. dubliniensis* may be implicated in IC beyond oropharyngeal involvement and document infections with this species in patients with hematologic malignancies, HIV/AIDS, and prior surgery. *C. lusitaniae*, previously found in patients with acute leukemia and neutropenia, was found to cause infections in patients <1 year of age, those with solid tumors and in surgical patients, whereas lack of infection due to *C. tropicalis* and *C. guilliermondii* in the pediatric age group is a new observation. Infection with multiple N-CA species occurred in 4.4% of patients and was associated with prior surgery and decreased 90-day survival. Examination of treatment choices reveals the prominent role of echinocandins over that of fluconazole in the treatment of N-CA species. However, evidence of de-escalation from an echinocandin to fluconazole was observed, especially in the *C. glabrata* cohort.

Limitations of the present study include differences in clinical practice patterns across the different centers, limited follow-up data, the inability to confirm the species identification, lack of antifungal susceptibility results as well as information on antifungal dosing practices. In addition, there was the inability to evaluate factors that may have contributed to successful outcomes and the inability to clearly distinguish between prophylactic and empirical therapy in sequential antifungal therapy as well as antifungal combination therapy. Furthermore, lack of denominator data prevented us from calculating incidence rates and the performance of risk factor analyses. Despite these limitations, the data collected by the PATH Alliance registry includes a very large number of patients with IC caused by N-CA species, with a broad spectrum of underlying conditions across a large number of centers in North America. Therefore this database will likely prove to be a significant asset in understanding the epidemiology, treatment, and outcomes of IC due to N-CA species.

## Supporting Information

Table S1 **Variation in frequency of non-** ***albicans*** **species of** ***Candida*** **by geographic region and participating hospitals.**
(DOCX)
