# Supplementary material for: Epidemiology and Outcomes of Invasive Candidiasis Due to Non-albicans Species of Candida in 2,496 Patients: Data from the Prospective Antifungal Therapy (PATH) Registry 2004–2008
Source: PLoS One. 2014 Jul 3;9(7):e101510. doi: 10.1371/journal.pone.0101510 (PMC4081561; doi:10.1371/journal.pone.0101510)
Supplement: Table S1 — Variation in frequency of non- albicans species of Candida by geographic region and participating hospitals. (DOCX) [file pone.0101510.s001.docx]

**Table S1. Variation in frequency of non-*albicans* species of *Candida* by geographic region and participating hospitals.**

|  | **Total no. (%) of patients with non-*albicans* *Candida* species** | | | | | | | | | | | |
| --- | --- | --- | --- | --- | --- | --- | --- | --- | --- | --- | --- | --- |
| **Region / hospital^*^** | **All patients** n = 5036 (100%) | **All N-CA** n = 2496 (49.6%) | ***C. glabrata*** n = 1159 (23.0%) | ***C. parapsilosis*** n = 616 (12.2%) | ***C. tropicalis*** n = 347(6.9%) | ***C. krusei*** n = 138(2.7%) | ***C. lusitaniae*** n = 41(0.8%) | ***C. dubliniensis*** n = 38(0.8%) | ***C. guilliermondii*** n = 9(0.2%) | **Other^†^** n = 22(0.4%) | **Unknown** n = 15(0.3%) | **Multiple species^§^** n = 111(2.2%) |
| Canada | 275 | 132 (48.0) | 53 (19.3) | 34 (12.4) | 24 (8.7) | 12 (4.4) | 1 (0.4) | 1 (0.4) |  |  |  | 7 (2.5) |
| H5 | 211 | 101 (47.9) | 41 (19.4) | 27 (12.8) | 19 (9.0) | 7 (3.3) |  | 1 (0.5) |  |  |  | 6 (2.8) |
| H6 | 64 | 31 (48.4) | 12 (18.8) | 7 (10.9) | 5 (7.8) | 5 (7.8) | 1 (1.6) |  |  |  |  | 1 (1.6) |
| Northeast | 1985 | 923 (46.5) | 427 (21.5) | 259 (13.0) | 129 (6.5) | 31 (1.6) | 11 (0.6) | 15 (0.8) | 4 (0.2) | 7 (0.4) |  | 37 (1.9) |
| H7 | 131 | 68 (51.9) | 18 (13.7) | 20 (15.3) | 10 (7.6) | 6 (4.6) | 3 (2.3) | 7 (5.3) |  | 1 (0.8) |  | 3 (2.3) |
| H10 | 465 | 252 (54.2) | 120 (25.8) | 65 (14.0) | 26 (5.6) | 8 (1.7) | 2 (0.4) | 3 (0.6) | 3 (0.6) | 4 (0.9) | 1 (0.2) | 20 (4.3) |
| H11 | 49 | 33 (67.3) | 19 (38.8) | 11 (22.4) | 1 (2.0) | 1 (2.0) |  |  |  |  | 1 (2.0) |  |
| H13 | 419 | 229 (58.2) | 110 (26.3) | 71 (16.9) | 26 (6.2) | 5 (1.2) | 2 (0.5) | 1 (0.2) | 1 (0.2) | 2 (0.5) |  | 11 (2.6) |
| H21 | 55 | 24 (43.6) | 12(21.8) | 5 (9.1) | 5 (9.1) | 1 (1.8) |  |  |  |  | 1 (1.8) |  |
| H22 | 659 | 204 (31.0) | 119 (18.1) | 46 (7.0) | 28 (4.2) | 10 (1.5) |  |  |  |  |  | 1 (0.2) |
| H25 | 207 | 113 (54.6) | 29 (14.0) | 41 (19.8) | 33 (15.9) |  | 4 (1.9) | 4 (1.9) |  |  |  | 2 (1.0) |
| South | 1483 | 845 (57.0) | 365 (24.6) | 203 (13.7) | 144 (9.7) | 59 (4.0) | 15 (1.0) | 4 (0.3) | 1 (0.1) | 4 (0.3) |  | 44 (3.0) |
| H3 | 253 | 127 (50.2) | 44 (17.4) | 37 (14.6) | 27 (10.7) | 7 (2.8) | 3 (1.2) | 2 (0.8) |  |  | 1 (0.4) | 6 (2.4) |
| H4 | 298 | 198 (66.4) | 116 (38.9) | 39 (13.1) | 19 (6.4) | 6 (2.0) | 1 (0.3) |  |  |  | 1 (0.5) | 16 (5.4) |
| H8 | 46 | 27 (58.7) | 3 (6.5) | 3 (6.5) | 10 (21.7) | 6 (13.0) | 2 (4.3) |  | 1 (2.2) | 1 (2.2) |  | 1 (2.2) |
| H9 | 55 | 16 (29.1) | 10 (18.2) | 1 (1.8) | 1 (1.8) | 3 (5.5) |  |  |  | 1 (1.8) | 1 (1.8) |  |
| H14 | 323 | 186 (57.6) | 88 (27.2) | 54 (16.7) | 31 (9.6) | 3 (0.9) |  |  |  |  |  | 10 (3.1) |
| H15 | 369 | 210 (56.9) | 81 (22.0) | 46 (12.5) | 31 (8.4) | 33 (8.9) | 6 (1.6) | 2 (0.5) |  | 2 (0.5) | 3 (0.8) | 6 (1.6) |
| H17 | 139 | 81 (58.3) | 23 (16.5) | 23 (16.5) | 23 (16.5) | 4 (2.9) | 3 (2.2) |  |  |  |  | 5 (3.6) |
| Midwest | 906 | 441 (48.7) | 244 (26.9) | 90 (9.9) | 37 (4.1) | 25 (2.8) | 8 (0.9) | 6 (0.7) | 1 (0.1) | 9 (1.0) | 4 (0.4) | 17 (1.9) |
| H1 | 34 | 18 (52.9) | 1 (2.9) | 7 (20.6) | 5 (14.7) | 2 (5.9) | 1 (2.9) |  |  | 1 (2.9) |  | 1 (2.9) |
| H16 | 151 | 73 (48.3) | 43 (28.5) | 15 (9.9) | 6 (4.0) | 2 (1.3) | 2 (1.3) | 2 (1.3) |  | 1 (0.7) |  | 2 (1.3) |
| H18 | 180 | 86 (47.8) | 39 (21.7) | 30 (16.7) | 6 (3.3) | 2 (1.1) | 2 (1.1) | 2 (1.1) |  | 3 (1.7) | 1 (0.6) | 1 (0.6) |
| H19 | 124 | 55 (44.4) | 29 (23.4) | 7 (5.6) | 3 (2.4) | 8 (6.5) |  | 2 (1.6) | 1 (0.8) |  |  | 5 (4.0) |
| H20 | 166 | 94 (56.6) | 58 (34.9) | 19 (11.4) | 7 (4.2) | 1 (0.6) | 2 (1.2) |  |  |  | 1 (0.6) | 6 (3.6) |
| H24 | 251 | 115 (45.8) | 74 (29.5) | 12 (4.8) | 10 (4.0) | 10 (4.0) | 1 (0.4) |  |  | 4 (1.6) | 2 (0.8) | 2 (0.8) |
| West | 387 | 155 (40.1) | 70 (18.1) | 30 (7.8) | 13 (3.4) | 11 (2.8) | 6 (1.6) | 12 (3.1) | 3 (0.8) | 2 (0.5) | 2 (0.5) | 6 (1.6) |
| H2 | 1 | 1 (100) |  |  |  | 1 (100) |  |  |  |  |  |  |
| H12 | 224 | 72 (32.1) | 29 (12.9) | 15 (6.7) | 9 (4.0) | 5 (2.2) | 5 (2.2) | 3 (1.3) | 2 (0.9) | 1 (0.4) | 2 (0.9) | 1 (0.4) |
| H23 | 162 | 82 (50.6) | 41 (25.3) | 15 (9.3) | 4 (2.5) | 5 (3.1) | 1 (0.6) | 9 (5.6) | 1 (0.6) | 1 (0.6) |  | 5 (3.1) |

^*^hospitals designated by the letter ‘H’; and a number: H1, H2 etc.

^†^Other species includes: *C. kefyr* (nine isolates), *C. famata* (four isolates), *C. rugosa* (three isolates)*, C. utilis* (two isolates) and one isolate each of *C. fennica*, *C. fermentati*, *C. lipolytica*, and *Torulopsis* spp.

^§^multiple species include *C. parapsilosis + C. glabrata* (n = 30), *C. tropicalis + C. glabrata* (n = 21)*, C. krusei + C. glabrata* (n = 8), *C. dubliniensis + C. glabrata* (n = 3), *C. lusitaniae + C. glabrata* (n = 4), other *Candida* spp. *+ C. glabrata* (n = 3), unknown *Candida spp. + C. glabrata* (n = 3), *C. guilliermondii + C. glabrata* (n = 2), *C. parapsilosis + C. krusei* (n = 5), *C. lusitaniae + C. krusei* (n = 2), *C. tropicalis + C. dubliniensis* (n = 1), *C. tropicalis + C. guilliermondii* (n = 1), *C. tropicalis + C. krusei* (n = 4), other *Candida* spp. + *C.* guilliermondii (n = 1), unknown *Candida* spp. + C*. dubliniensis* (n = 1), *C. glabrata + C. krusei + C. lusitaniae* (n = 1), *C. dubliniensis + C. glabrata + C. guilliermondii* (n = 1).
